# Quantitative, High-Throughput Assays for Proteolytic Degradation of Amylin

**DOI:** 10.3390/mps3040081

**Published:** 2020-11-24

**Authors:** Caitlin N. Suire, Monica K. Brizuela, Malcolm A. Leissring

**Affiliations:** 1 Institute for Memory Impairments and Neurological Disorders, University of California, Irvine (UCI MIND), Irvine, CA 92697, USA; csuire@uci.edu (C.N.S.); mkbrizue@uci.edu (M.K.B.); 2 Department of Neurobiology and Behavior, University of California, Irvine, CA 92697, USA

**Keywords:** amylin, insulin-degrading enzyme, proteolytic degradation, high-throughput screening, type-2 diabetes mellitus

## Abstract

Amylin is a pancreatic peptide hormone that regulates glucose homeostasis but also aggregates to form islet amyloid in type-2 diabetes. Given its role in both health and disease, there is renewed interest in proteolytic degradation of amylin by insulin-degrading enzyme (IDE) and other proteases. Here, we describe the development and detailed characterization of three novel assays for amylin degradation, two based on a fluoresceinated and biotinylated form of rodent amylin (fluorescein-rodent amylin-biotin, FrAB), which can be used for any amylin protease, and another based on an internally quenched fluorogenic substrate (FRET-based amylin, FRAM), which is more specific for IDE. The FrAB-based substrate can be used in a readily implemented fluorescence-based protocol or in a fluorescence polarization (FP)-based protocol that is more amenable to high-throughput screening (HTS), whereas the FRAM substrate has the advantage of permitting continuous monitoring of proteolytic activity. All three assays yield highly quantitative data and are resistant to DMSO, and the FRAM and FP-based FrAB assay are ideally suited to HTS applications.

## 1. Introduction

Amylin, also known as islet amyloid polypeptide, is a peptide hormone that is stored in pancreatic beta-cells and co-secreted with insulin in response to many factors, including glucose, arginine, and fatty acids [1]. Amylin plays a role in inhibiting glucagon secretion and delaying gastric emptying as well as modulating other aspects of food processing and satiety [2]. Human amylin is highly aggregation-prone, and in type-2 diabetes mellitus (T2DM) frequently forms insoluble deposits known as islet amyloid [3]; moreover, potentially pathological deposits of human amylin also occur in the brain in aging and Alzheimer’s disease [4,5]. Despite its physiological and pathophysiological significance, very little is known about the catabolism of amylin, and appropriate methods and experimental tools for studying this topic are greatly needed.

Amylin is a 37-residue peptide with a highly conserved sequence that is present in all mammals [6]. To be fully active, two major post-translational modifications are required: a C-terminal amide (Tyr37) and a disulfide bond between two cysteine residues found at positions 2 and 7 [6]. Amylin can be hydrolyzed by several proteases, including insulin-degrading enzyme (IDE) [7,8,9,10,11] and beta-site APP-cleaving enzyme 2 (BACE2) [12]. As is true for other amyloidogenic substrates, such as the amyloid β-protein (Aβ), it is likely that multiple amylin proteases exist that work in concert to regulate steady-state amylin levels [13]. IDE in particular is of special interest because it has been shown to regulate amylin levels in vivo [7], and also because genetic variations in and around the *Ide* gene have been linked to T2DM [14,15]. Intriguingly, IDE also degrades several other peptide hormones relevant to glucose homeostasis, including insulin and glucagon [7,8]; consequently, there is growing interest in disentangling the different ways that IDE activity can regulate glucose homeostasis, in particular by developing substrate-selective inhibitors such as the insulin-selective IDE inhibitor recently developed by Maianti and colleagues [16]. Despite growing interest in amylin degradation, only expensive and cumbersome assays based on ELISA and HPLC are currently available. To address this need, we describe here the development and characterization of three novel assays for amylin degradation that are inexpensive, easy to use, and highly quantitative.

## 2. Materials and Methods

### 2.1. Materials

Substrate 1 ([5-FAM]KCNTATCATQRLANFLVHSSNNFGAILSSTNVGSNTY[Lys(Ahx-biotin)]-amide; 99% purity), substrate 2 ([Lys(Ahx-biotin)]CNTATCATQRLANFLVRSSNNL-GPVLPPTNVGSNT[Lys(FITC)]-amide; 97% purity), substrate 3 (fluorescein-rodent amylin-biotin, FrAB; [5-FAM]KCNTATCATQRLANFLVRSSNNLGPVLPPTNVGSNTY[Lys(Ahx-biotin)]-amide; 97% purity), and substrate 4 (FRET-based amylin, FRAM; KCNTATCATQR[Lys(DABCYL)]ANF-LVR[Glu(EDANS)]SNNLGPVLPPTNVGSNTY; 96% purity) were synthesized by Thermo Fisher Scientific, Inc. (Waltham, MA, USA) via solid-phase peptide synthesis, each with a disulfide bond between Cys2 and Cys7 (Table 1). Substrates were dissolved in DMSO as 1 mM stocks as calculated from the manufacturer-provided weight of lyophylized peptide and its MW, taking into account purity levels. Egg white avidin; neutravidin agarose beads; and Corning black, 384-well, low-volume, round-bottom, non-binding surface (NBS) microplates were purchased from Thermo Fisher Scientific, Inc. (Whatham, MA, USA). PlateOne 96-well round-bottom polypropylene plates were purchased from USA Scientific (Ocala, FL, USA). Omnipur BSA, Fraction V (lacking biotin), and PBS (reconstituted from tablets) were purchased from Sigma-Aldrich, Inc. (Saint Louis, MO, USA). Recombinant human IDE was generated and purified as described [17]. The specific activity of recombinant IDE was verified to be within 5% of the value determined from protein concentration (quantified by nanodrop).

### 2.2. Amylin Degradation Assays

All assays were carried out in PBS supplemented with 0.05% biotin-free BSA, typically using 1 nM recombinant IDE and compounds at various concentrations in 1% DMSO. To ensure homogeneity of solution concentrations, we mixed all solutions well by gentle rocking on a nutator for ≥10 min at room temp (21–23 °C), and dispensed them manually using 16-channel pipettors fitted with 20-µL FinnTip pipette tips. For experiments conducted in 384-well plates, or transfers thereto, mixing was performed by ≥3 rounds of trituration after dispensing of the final solution. For avidin-agarose precipitation (AP)-based assays, 3× IDE (200 µL) and 3× compound or DMSO-only (200 µL) were combined in 1.5-mL centrifuge tubes and allowed to equilibrate at room temperature for ≥5 min. Reactions were initiated by addition of 3× FrAB substrate (200 µL), typically at 100 nM final concentration, except where indicated. After the appropriate incubation period, reactions were terminated by addition of 4× AP Stop Buffer (200 µL; PBS/0.05% biotin-free BSA containing a final concentration of 2 mM 1.10-phenanthroline and 10-fold molar excess avidin agarose beads). After rocking on a nutator for 20 min to allow biotin–avidin interactions to complete, tubes were centrifuged for 10 min at 15,000× *g*. After we carefully removed the tubes from the centrifuge to avoid disturbing the pelleted avidin-agarose, the supernatant was transferred to low-volume 384-well microplates (30 µL/well), then fluorescence (λ_ex_ = 485 nm, λ_em_ = 515 nm) was read on a multilabel plate reader (SpectraMAX, Molecular Devices, San Jose, CA, USA).

For fluorescence polarization (FP)-based assays, reactions were typically performed directly in low-volume 384-well microplates. To that end, 3× IDE solution (8 µL) was combined with 3× compound or DMSO (8 µL) and allowed to equilibrate for ≥5 min at room temperature. Reactions were initiated by addition of 3× FrAB substrate (8 µL), typically at 100 nM final concentration, except where indicated. After the appropriate incubation period, reactions were terminated by addition of 4× FP Stop Buffer (8 µL; PBS/0.05% biotin-free BSA containing a final concentration of 2 mM 1,10-phenanthroline and 10-fold molar excess avidin (monomer)), triturated ≥3 times to ensure even mixing. As determined empirically in previous work [18], biotin–avidin interactions proceeded to completion within 2 min, but reactions were allowed to incubate for ≥ 10 min at room temperature prior to reading to ensure complete binding. Fluorescence polarization (λ_ex_ = 485 nm, λ_em_ = 515 nm) was read on a multilabel plate reader (SpectraMAX M5^e^, Molecular Devices, San Jose, CA, USA) using the default fluorescence polarization protocol, with the g-factor value maintained at 1.0. high-throughput screening (HTS) being performed manually using a 16-channel pipettor.

For fluorescence-dequenching assays, reactions were typically performed directly in low-volume 384-well microplates, and because the measurements could be monitored continuously, there was no need to stop the reactions. To that end, 3× IDE solution (10 µL) was combined with 3× compound or DMSO (10 µL) and allowed to equilibrate for ≥5 min at room temperature. Reactions were initiated by addition of 3× FrAB substrate (10 µL), typically at 100 nM final concentration. Fluorescence (λ_ex_ = 336 nm, λ_em_ = 490 nm) was monitored repeatedly at frequent intervals (e.g., 30 s) on a multilabel plate reader (SpectraMAX, Molecular Devices, San Jose, CA, USA) using the default fluorescence measurement protocol.

### 2.3. Data Analysis

For all assays, results were normalized to data from negative (0% hydrolysis, no IDE) and positive (100% hydrolysis, excess IDE) controls and, where appropriate, expressed as percent hydrolysis. In the case of AP-based assays, percent hydrolysis was a linear function of the percent maximal change in relative fluorescence (RFU; %ΔRFU) between positive and negative controls. In the case of FP-based assays, percent maximal change in millipolarization units (mP; %ΔmP) was first calculated, then converted to percent hydrolysis using the following formula (Equation (1)):

*Y* = 0.5553*X* + 0.004409*X*^2^ (1)

where *X* is %ΔmP and Y is percent hydrolysis. In the case of continuously monitored fluorescence-dequenching assays with the FRAM substrate, the slopes of ΔRFU as a function of time were obtained from linear portions of progress curves using the manufacturer’s software (SoftMax Pro v5.0, Molecular Devices, San Jose, CA, USA), then normalized to negative controls lacking IDE and positive controls containing DMSO only. For endpoint assays using the FRAM substrate, RFU values are reported directly.

### 2.4. Numerical Analyses

Linear and non-linear curve fitting was conducted using Prism 8.0 for MacOS (GraphPad Software, LLC, San Diego, CA, USA). Equation (1) was derived by fitting a second-order polynomial, constrained to go through *X* = *Y* = 0, to normalized FP data for known quantities of hydrolyzed FRAM, where percent hydrolysis was plotted as a function of %ΔmP. For dose–response curves, IC_50_s and EC_50_s were obtained by from sigmoidal curves fitted to linear response as a function of log concentration inhibitor or avidin. IC_50_s were converted to K_i_ values using the Cheng–Prusoff equation [19]. The reliability of the different assays was assessed by calculating *Z*’ factor values [20], according to the following equation (Equation (2)):

(2) $$Z^{\prime }=1-\left[\frac{3\left(\sigma_{\mathrm{HI}}+\sigma_{\mathrm{LO}}\right)}{\mu_{\mathrm{HI}}-\mu_{\mathrm{LO}}}\right]$$

where σ*_HI_* and σ*_LO_* correspond to the standard deviations and μ*_HI_* and μ*_LO_* the means of the high and low readings (mP or RFU), respectively.

## 3. Results

### 3.1. Development and Characterization of AP and FP-Based Assays for Amylin Degradation

**Figure 1:**
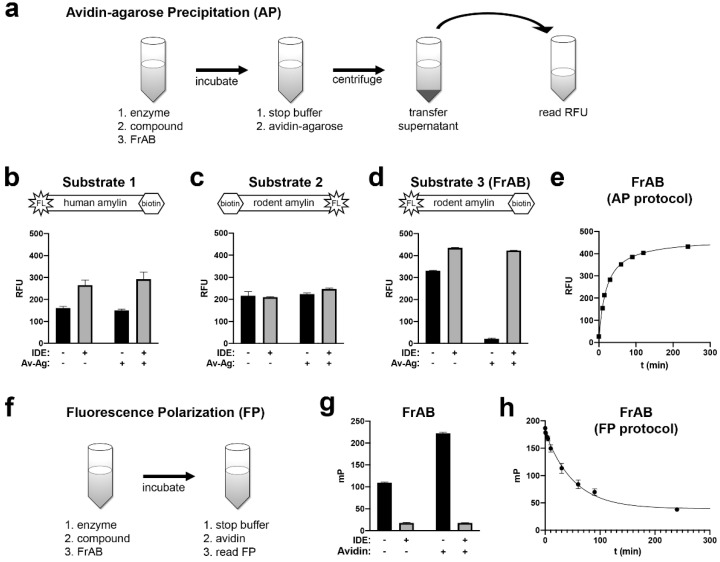
Testing of various fluoresceinated and biotinylated amylin substrates for suitability to the avidin-agarose precipitation (AP) and fluorescence polarization (FP) protocols. (**a**) Cartoon showing a generalized overview of the AP protocol. Note the large number of steps, including centrifugation and liquid transfer. (**b**) Cartoon of the structure of substrate 1 (*top*) and relative fluorescence (RFU) obtained for uncleaved (-IDE) and cleaved (+ IDE) Substrate 1 (1 μM) before (-) or after (+) avidin-agarose (Av-Ag) precipitation (*bottom*). (**c**,**d**) Similar graphs for substrate 2 (**c**) and substrate 3 (**d**). Note that the uncleaved versions of substrates 1 and 2 were not precipitated by Av-Ag, as expected, whereas uncleaved substrate 3 was. Note also that the fluorescence of fully cleaved substrate 3 remained constant before and after AP. (**e**) Typical progress curve observed with the AP-based method using 1 μM fluorescein-rodent amylin-biotin (FrAB). (**f**) Cartoon showing a generalized overview of the FP protocol. Note that this is a “mix-and-measure” method (requiring only addition steps), making it well suited for high-throughput screening (HTS). (**g**) Changes in FP (expressed in millipolarization units, mP) obtained with uncleaved (- IDE) versus fully hydrolyzed (+ IDE) FrAB (1 μM) in the absence (−) or presence (+) of avidin (4 μM, corresponding to 16 µM biotin-binding sites). (**h**) Progress curve observed with the FP protocol using 3 μM FrAB.

In previous work, we developed robust proteolytic degradation assays for Aβ [18] and glucagon [21] on the basis of synthetic versions of these substrates containing fluorescein at the N‑terminus and biotin (in the form of Lys(Ahx-biotin)) at the C-terminus. This general approach enables a facile method for detecting hydrolysis, which we call avidin-agarose precipitation (AP). Briefly, hydrolysis can be readily quantified by removing the intact (biotinylated and fluoresceinated) substrates with avidin-agarose, then quantifying the remaining (fluoresceinated only) proteolytic fragments by fluorescence. This approach has the advantage of being readily implementable in any laboratory equipped with a fluorescence reader. Although convenient, the AP protocol requires centrifugation and liquid transfer steps that make it less amenable to high-throughput or robotized applications (Figure 1a).

On the assumption that this approach would be readily generalizable to amylin, we synthesized N-terminally fluoresceinated and C-terminally biotinylated human amylin, complete with an internal disulfide bond between Cys2 and Cys7, dubbed substrate 1 (Figure 1b; Table 1). As has been observed with similar substrates [18,21], complete hydrolysis of substrate 1 by excess IDE resulted in a modest increase in fluorescence relative to uncleaved substrate 1 (Figure 1b). Surprisingly, however, uncleaved substrate 1 could not be precipitated by avidin-agarose, apparently because the biotin moiety was inaccessible (Figure 1b). Because the C-terminal portion of human amylin is the most aggregation-prone region of the peptide [6], we hypothesized that strong secondary structure in this area may have rendered the biotin moiety inaccessible to productive interactions with avidin. We therefore synthesized two new substrates, in this case on the basis of rodent amylin, which is far less prone to aggregation and secondary structure formation than the human peptide [1]. In one version, substrate 2 (Figure 1c; Table 1), we swapped the positions of the fluorescein and biotin groups, while in the other, substrate 3 (Figure 1d; Table 1), we maintained their placement at the N‑ and C-termini, respectively. Surprisingly, substrate 2, in which Lys (Ahx-biotin) was substituted for the endogenous lysine residue at position 1 (Table 1), also could not be precipitated successfully by avidin-agarose (Figure 1c), in this case likely because the biotin moiety was sterically hindered by the presence of the disulfide bond near the N-terminus of amylin. In marked contrast, substrate 3 behaved as expected—intact substrate was readily precipitated by avidin-agarose, and hydrolyzed substrate yielded a strong fluorescence signal after precipitation (Figure 1d). On the basis of these preliminary results, we elected to use substrate 3 for downstream assay development, renaming it FrAB (fluorescein-rodent amylin-biotin). As expected, hydrolysis of FrAB could be readily monitored using the AP protocol, with hydrolysis resulting in a steady increase in fluorescence signal as a function of time in the presence of low quantities of IDE (Figure 1e).

Though easy to implement, the AP protocol is not ideal for high-throughput or robotized applications due to the requisite centrifugation and liquid transfer steps (Figure 1a). Fortunately, hydrolysis of FrAB can also be monitored via a fluorescence polarization (FP)-based method that is more amenable to high-throughput use. Briefly, when excited by plane-polarized photons, stationary fluorescent molecules emit lower energy photons that are polarized in the same plane as (or at a fixed angle to) the absorbed photons [22]. When in solution, fluorescently tagged molecules rotate, or tumble, at a rate that is inversely proportional to their size. Because there is a slight delay between the absorption and emission of individual photons, the emitted photons will be depolarized to a greater extent by smaller fragments, which tumble quickly, versus larger fragments, which tumble more slowly. Hydrolysis of FrAB will result in shorter fragments that will tumble (slightly) more quickly than the intact substrate. However, by virtue of biotin at the C-terminus, the rotation of uncleaved substrate can be slowed significantly further by addition of avidin, a 64-kDa tetramer that is considerably larger than the substrate. The cleaved, fluoresceinated fragments, by contrast, will continue to tumble unimpeded, and thus FP can be used to quantify the degree of cleaved versus uncleaved substrate. When using FP, the general protocol is significantly streamlined, involving only addition steps (Figure 1f), thus making it a true “mix-and-measure” assay [22].

To assess the suitability of FrAB to the FP protocol, we measured the degree of depolarization (measured in millipolarization units, mP) in uncleaved versus fully cleaved substrate in the absence or presence of excess avidin. As seen in Figure 1g, fully cleaved FrAB depolarized light (reflected in lower mP values) significantly more than uncleaved substrate. Not only was substantially less depolarization observed with the intact substrate alone, but the depolarization was reduced even further by the addition of avidin (Figure 1g). As expected from the preceding results, hydrolysis of FrAB as a function of time could be readily monitored using the FP protocol (Figure 1h).

**Figure 2:**
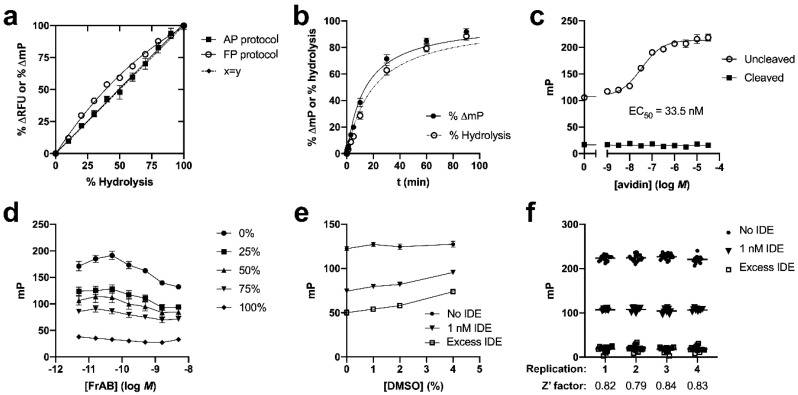
Characterization of the FP-based amylin degradation assay. (**a**) Relationship between percent signal change and percent hydrolysis of FrAB for the FP- and AP-based methods. Note that the relationship was linear for the AP protocol, but somewhat nonlinear for the FP protocol. Data for the latter were consequently used to generate a formula for conversion of raw %ΔFP data to percent hydrolysis for quantitative analyses (Equation (1) in *Materials and Methods*). Data are mean ±SEM for three independent experiments. (**b**) Progress curve obtained with the FP protocol showing raw %ΔFP values (*solid line*) and the same data converted to percent hydrolysis (*dashed line*) using the equation derived from the data in (**a**) (Equation (1)). (**c**) Effect of avidin (monomer) concentration on measured mP values for a fixed concentration of cleaved and uncleaved FrAB (100 nM). Note that fully cleaved fragments were unaffected by avidin, as seen in Figure 1g. Data are mean ± SEM for four independent experiments. (**d**) Relationship between raw mP values and FrAB concentration for different degrees of hydrolysis. Data are mean ± SEM for three independent experiments. (**e**) Effect of DMSO concentration on performance of the FP-based assay. Data are mean ± SEM for four independent experiments. (**f**) Raw data and calculated *Z*’ factor values for four independent experiments conducted in high-throughput (384-well) format on separate days. Average *Z*’ factor across replicates was 0.82.

Next, we conducted a series of experiments to more fully characterize the FrAB substrate, particularly for FP-based applications. Raw FP values do not always change linearly with changes in hydrolysis, and thus it was necessary to investigate and quantify this relationship. To do so, we combined varying amounts of fully cleaved substrate and intact substrate so as to obtain different fixed percentages of hydrolyzed FrAB, which were then quantified using the AP and FP protocols. As shown in Figure 2a, with the AP protocol, the percent change in fluorescence (%ΔRFU) tracked essentially linearly with percent hydrolysis. In contrast, in terms of the FP protocol, the percent change in mP (%ΔmP) exhibited a non-linear relationship, with %ΔmP overestimating the amount of hydrolysis for values <40% (i.e., corresponding to the linear range of most progress curves), as has been seen previously with other substrates using the same protocol [18,21]. We used these results to calculate a correction factor (Equation (2)) for converting %ΔmP to percent hydrolysis. (Note: due to differences in FP-capable readers, it would be advisable to conduct a similar experiment to calculate the correction factor specific to the machine in use.) Figure 2b shows progress curves expressed in units of both raw %ΔmP and percent hydrolysis after conversion with the correction formula. Although the differences appear modest, they are particularly important when determining the initial velocity (*v*_o_) of reactions, and thus the use of this correction formula is advised for quantitative applications.

Avidin (and avidin-agarose) is an expensive reagent, and thus it is valuable to establish the minimum avidin/FrAB ratio necessary for reliable results. To that end, we added varying amounts of avidin to a fixed quantity (100 nM) of both cleaved and uncleaved FrAB and assessed the results using the FP protocol. As illustrated in Figure 2c, mP values increased with increasing avidin (monomer) concentration, with an apparent EC_50_ of 33.5 ± 7.5 nM (*n* = 4), in good agreement with the known concentration of FrAB (100 nM). Notably, the increase in mP values reached a plateau, beginning at ≈1 µM avidin, with no significant additional effect observed at higher concentrations (Figure 2c). Accordingly, we recommend that a minimum avidin/FrAB ratio of ≈10:1 be used in practice. It is notable that fully cleaved FrAB was unaffected even by very high concentrations of avidin (Figure 2c).

We also assessed how raw mP values vary as a function of FrAB concentration. To this end, we prepared different percentages of hydrolyzed FrAB (0%, 25%, 50%, etc.) and quantified mP values using the FP protocol, being careful to use a constant 10:1 ratio between (monomeric) avidin and FrAB. As illustrated in Figure 1d, the raw mP values varied somewhat when assessed across a broad range of concentrations spanning several orders of magnitude. Nevertheless, only minimal changes were observed between semilog differences in concentration. These results imply that the assay is fairly impervious to relatively modest changes in absolute concentration of substrate, as might accrue due to pipetting errors, likely due to the fact that FP is a ratiometric measure [22].

To assess the extent to which the assay could tolerate different DMSO concentrations, we used the FP protocol to quantify raw mP values for a fixed amount of FrAB (100 nM), including uncleaved, fully cleaved, and partially cleaved substrate, in the presence of varying amounts of DMSO. As shown in Figure 2e, although the absolute mP values obtained varied modestly between different concentrations of DMSO, excellent separation was observed across a range of DMSO concentrations as high as 4%, suggesting that the assay is highly tolerant to DMSO.

To assess the reliability and reproducibility of the assay in a more quantitative manner, we conducted four independent experiments, executed on separate days, wherein we quantified a large number of replicates of uncleaved, fully cleaved, and partially cleaved FrAB. We then calculated the *Z*’ factor values, a measure frequently used to assess the robustness of HTS assays [20], using the formula in Equation (2). As illustrated in Figure 2f, we obtained high values for all replicates, with an average *Z*’ value of 0.82.

**Figure 3:**
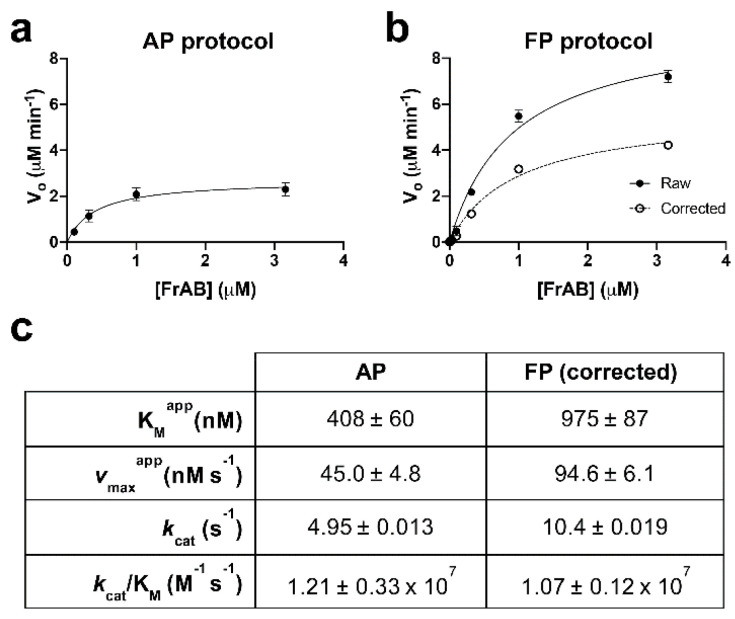
Kinetics of FrAB degradation by IDE assessed using the AP and FP protocols. Results obtained using the AP (**a**) and FP (**b**) protocols, showing initial velocity (*v_O_*) plotted as a function of FrAB concentration. Data from the FP-based assay are plotted both in terms of raw percent change in mP (*solid line*) and those data converted to percent hydrolysis using Equation (1) (*dashed line*). Note that uncorrected, raw data significantly overestimate the *v_max_* of the reaction. Data are mean ± SEM for four independent data points grouped together and fitted to individual hyperbolae only for the purposes of these graphs. (**c**) Kinetic parameters derived from these results. Note that these data reflect the means ± SEM calculated separately for each of four independent replications for each protocol.

We next used the AP and FP protocols to determine the kinetic parameters of FrAB degradation by IDE. To that end, time courses were conducted using varying concentrations of FRAM and identical amounts of IDE (1 nM). From these data, the initial velocity (*v*_o_) of each reaction was calculated and plotted as a function of FRAM concentration. Figure 3 shows the resulting data, plotting the means and standard errors for each substrate concentration from four independent experiments using the AP (Figure 3a) and FP (Figure 3b) protocols. For the FP protocol, we plotted both the raw data (derived from uncorrected %ΔmP data) and the same data after correction to percent hydrolysis. We note that no correction was necessary for data obtained with the AP protocol, because the relationship between percent hydrolysis and percent RFU was linear (Figure 2a). The Michaelis–Menten constant (K_M_), the turnover number (*k*_cat_), and other kinetic parameters obtained from each of four independent replications for each of the assays are shown in Figure 3c. Note that the absolute values for all parameters were in good agreement between the AP and FP protocols (Figure 3c) and were similar to the values obtained for other IDE substrates [17,18,21,23].

**Figure 4:**
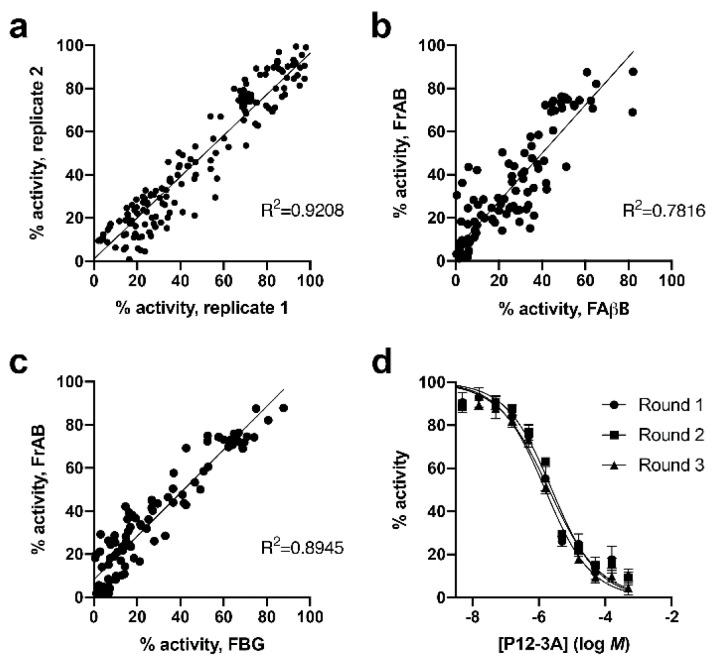
Performance of the FP-based amylin degradation assay in HTS format. (**a**–**c**) IDE activity in the presence of ≈100 IDE inhibitors (160 µM in 1% DMSO) screened using the FP protocol with FrAB alone, one replication versus another, conducted on separate days (**a**); FrAB versus the Aβ substrate, FAβB (**b**); and FrAB versus the glucagon substrate, FBG (**c**). Note that (**a**) depicts the correlation between singlicate measurements obtained in separate replications conducted on separate days, whereas (**b**) and (**c**) depict the correlations between the average of duplicate measurements for each substrate and each IDE inhibitor. (**d**) Dose–response curves obtained for IDE inhibitor P12‑3A using the FP-based amylin degradation assay. See text for derived K_i_ values. Data are mean ± SEM for three independent experiments.

To establish the utility of the FP-based amylin degradation assay in various real-world applications, we conducted a range of experiments. First, we screened a collection of IDE inhibitors at different concentrations in high-throughput (384-well) format. Compounds were tested in duplicate, and comparison of the corrected percent hydrolysis for one singlicate read versus another, conducted on separate days, showed good agreement (Figure 4a; *R*^2^ = 0.92 by linear regression). We then compared the results obtained from the FP protocol conducted with FrAB with those obtained using similar FP-based assays for Aβ [18] (Figure 4b) and glucagon [21] (Figure 4c). Keeping in mind that inhibitors of IDE frequently exhibit substrate-specific differences in potency [16,24] (this being a key rationale for the development of this assay), the correlations between assays were reasonably strong. Finally, we conducted three independent dose–response curves on a well-characterized peptidic inhibitor of IDE, P12-3A [25]. As shown in Figure 4d, these assays yielded IC_50_ values in close agreement with one another, resulting in calculated K_i_ values of 1.85 ± 0.24 µM, in excellent agreement with published values for the inhibition of several other IDE substrates [25].

### 3.2. Development and Characterization of a Fluorescence-Dequenching Amylin Degradation Assay

In principle, FrAB should be able to detect the activity of any amylin protease, because the design is such that hydrolysis of any peptide bond within the amylin sequence can be detected. At the same time, the AP and FP protocols using FrAB are end-point assays, requiring that the reactions be terminated before activity can be measured, and/or requiring specialized equipment, which can be disadvantageous. Given the demonstrated in vivo relevance of IDE to amylin proteostasis [7], we sought to develop a continuous assay specifically suited to monitoring amylin degradation by IDE. To that end, we used the co-crystal structure of human IDE in complex with amylin (PDB 3HGZ) [26] to guide the design of an internally quenched amylin peptide. In this co-crystal structure, which uses a proteolytically inactive form of IDE, amylin is positioned such that hydrolysis would be mediated between Phe15 and Leu16. Using the crystal structure as a guide, we elected to introduce a fluorophore-quencher FRET pair flanking these residues. In particular, we introduced the fluorophore/donor 5-((2-aminoethyl)amino)naphthalene-1-sulfonic acid (EDANS) at position 19 (in the form of Glu(EDANS)) and the quencher/acceptor 4-(dimethylaminoazo)benzene-4-carboxylic acid (DABCYL) at position 12 (in the form of Lys(DABCYL)). As was done for other substrates, we elected to incorporate this FRET pair in rodent amylin containing an internal disulfide bond between Cys2 and Cys7 (Table 1).

**Figure 5:**
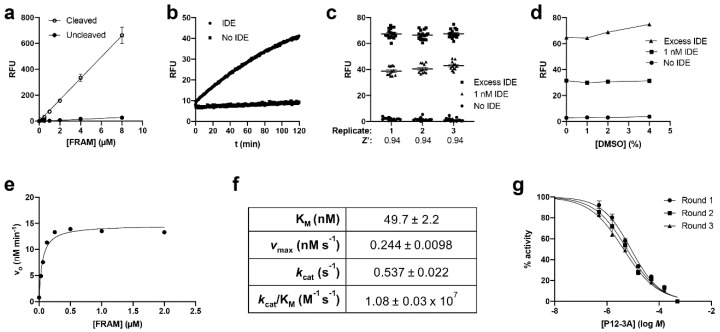
Characterization of the FRET-based amylin (FRAM) substrate for use as a fluorescence-dequenching amylin degradation assay. (**a**) FRAM substrate at various concentrations, uncleaved and fully cleaved by IDE. Note the linearity of the relationship between RFU and [FRAM] and the large (≈25-fold) fluorescence increase in hydrolyzed vs. non-hydrolyzed substrate. Data are mean ± SEM for 4–8 measurements per condition. (**b**) Typical progress curve obtained with FRAM (1 µM) in the absence or presence of IDE (0.1 nM). Note that the assay can be monitored continuously, without the need to terminate the reaction before reading. (**c**) Raw RFU data and *Z*’ factor values calculated therefrom for three replications conducted on separate days. (**d**) Influence of DMSO on the FRAM-based assay. Data are mean ± SEM for four independent experiments. (**e**) Initial velocity plotted as a function of FRAM concentration. Data are mean ± SEM for four independent experiments. (**f**) Kinetic data derived from the experiments in (**e**). (**g**) Dose–response curves obtained for inhibition of IDE by P12-3A obtained on separate days using the FRAM-based fluorescence dequenching amylin degradation assay. Note the similarity with the data obtained with FrAB and the FP protocol (Figure 4d). For each replication, data are mean ± SEM for four readings.

The resulting peptide, substrate 4 or FRAM (FRET-based amylin; Table 1), proved to be an effective IDE substrate. Cleavage of FRAM by IDE (or trypsin, not shown) resulted in ≈25-fold increases in fluorescence relative to uncleaved substrate across a range of concentrations (Figure 5a), indicating that effective internal quenching was occurring. As expected, because hydrolysis of FRAM could be monitored continuously, the resulting progress curves were well behaved (Figure 5b). *Z*’ value measurements obtained for 1 µM FRAM in independent runs conducted on separate days (Figure 5c) were consistently high, averaging 0.94. The reaction was not negatively impacted by DMSO concentrations up to 4% (Figure 5d). Taken together, these results suggest FRAM is well suited to HTS applications.

To complete the characterization of the FRAM substrate, we determined the kinetics of its degradation by IDE (Figure 5e). To our surprise, the K_M_ of FRAM for IDE was remarkably low (50 ± 2.2 nM; Figure 5f), significantly lower than the K_M_ values for FrAB determined using the AP and FP protocols (cf. Figure 3c). The *k*_cat_ of FRAM hydrolysis by IDE was correspondingly lower (0.54 ± 0.02 s^−1^) than the *k*_cat_ values obtained for the FrAB substrate (4.95 and 10.4 s^−1^, respectively, for the AP and FP protocols), such that the catalytic efficiency (*k*_cat_/K_M_) of hydrolysis of the FrAB and FRAM substrates by IDE was comparable for all three assays (≈1 × 10^7^ M^−1^ s^−1^; cf. Figure 3c and Figure 5f). Finally, to assess the utility of the FRAM substrate for real-world applications, we used it to conduct dose–response curves for the IDE inhibitor P12-3A (Figure 5g). In three experiments conducted on separate days, we obtained IC_50_ values that were close to one another (6.2 ± 1.2 µM). When converted to K_i_ values using the Cheng–Prusoff equation [26], we obtained values (K_i_ = 0.294 ± 0.056 µM) that were somewhat lower than published values [25] or what was obtained using the AP and FP protocols (Figure 4d). These discrepancies are discussed in the next section.

## 4. Discussion

In the present work, we developed and characterized three novel assays for amylin degradation. Two assays were based on FrAB, an N-terminally fluoresceinated and C-terminally biotinylated rodent amylin substrate. The first assay relies on the AP protocol, which can be easily implemented in any laboratory equipped with a fluorescent plate reader. The second FrAB-based assay utilizes an FP-based protocol; this one is better suited for HTS applications because it is a pure “mix-and-measure” assay [22], but it requires specialized equipment capable of reading FP or fluorescence anisotropy. Significantly, the FrAB-based assays, although thus far only tested with IDE, should be appropriate for assaying hydrolysis of any amylin protease.

The development of successful assays for amylin degradation based on the AP and FP protocols was more complicated than expected. Two of the substrates we tested showed no ability to bind to avidin. Given that several other successful assays based on this approach have been developed without complications [18,21], this was an unexpected outcome. While we can only speculate as to the mechanisms involved, for substrate 1, which was based on the aggregation-prone human form of amylin, it seems likely that the inability of the C-terminal biotin moiety to make interactions with avidin pertained to its known tendency to form strong secondary structures, particularly beta strands [27]. In the case of substrate 2, which was based on the far less aggregation-prone rodent version of amylin, it would appear the disulfide bond between Cys2 and Cys7, being very near to the N‑terminus, likely placed the biotin moiety in a position that was unfavorable to avidin binding. We note that it might be possible to overcome this problem by adding a reducing agent to the stop buffer along with avidin or avidin-agarose; however, substrate 2 has another disadvantage, namely, that the fluorescein isothiocyanate (FITC) molecule is attached to the peptide via a linker, rather than directly linked to the peptide back bone, as is the case for the N-terminal fluorescent moieties. Attachment via a linker makes this substrate liable to the “propeller effect”, wherein the fluorescent moiety rotates rapidly on this linker, independently of the status of the remainder of the protein, thus depolarizing light more than it ordinarily would [22]. Given our success with FrAB, there is no need to pursue substrate 2 further, but it reinforces the desirability of attaching the fluorescent group rigidly, which in the case of fluorescein is most easily done at the N-terminus of peptides.

We also developed a FRET-based fluorescence dequenching assay, which was successful in many respects, but about which some caution is warranted. First, the particular placement of the fluorescing EDANS and quenching DABCYL groups was designed to be optimal for a known cleavage site hydrolyzed by IDE [26]. This particular placement may not be ideal for other amylin proteases, depending on where they cleave the peptide. Secondly, whereas the FrAB-based AP and FP protocols both yielded kinetic parameters close to one another and also similar in magnitude to those obtained for other IDE substrates [17,18,21,28], the kinetic parameters of FRAM hydrolysis by IDE differed substantially. At first site, it might seem relevant that the kinetic parameters for FrAB (and similar IDE substrates) are technically “apparent” values only, because IDE can cut amylin at multiple sites within the peptide [26], whereas the FRAM substrate targets just one cleavage site. However, if IDE exhibited a K_M_ substantially stronger for one cleavage event versus another, the FrAB-based assays would nevertheless detect this preferred cleavage event, and the K_M_ would be closer to that obtained with FRAM. Rather, the very low K_M_ value (≈50 nM) obtained for FRAM hydrolysis by IDE, as well as the much slower *k*_cat_, could more likely be interpreted to suggest that the EDANS and DABCYL disrupt normal binding—perhaps making the substrate comparatively “sticky” relative to unmodified amylin, thus affecting both the affinity and the off rates of substrate and/or products. Consistent with this explanation, using FRAM, our estimates for the K_i_ value of IDE inhibitor P12-3A deviated by more than an order of magnitude from the results with the FrAB-based assays and with previous measurements with other IDE substrates [21] after conversion using the Cheng–Prusoff equation [26]. Given these observations, some caution is warranted when using the FRAM-based assay—the substrate might be useful for primary HTS screens, but for more quantitative analyses, the FrAB-based amylin degradation assays are preferred.

Overall, these novel assays offer significant advantages over existing amylin degradation assays in terms of cost and suitability for HTS. Amylin ELISAs, for example, are only commercially available in 96-well format and require numerous liquid handling steps that preclude their use in HTS assays. In addition to their high cost (several USD per sample), it is necessary to also purchase synthetic amylin peptide for degradation assays, further increasing the overall cost. Finally, for many applications, there is a need to dilute the samples prior to application to the ELISA plate, further increasing the liquid handling steps and the possibility for errors (in our hands, ELISA-based amylin degradation assays never yielded *Z*’ factor values exceeding 0.3). In marked contrast, 1 mg of the FrAB or FRAM substrates is all that is required to carry out ≈20,000 reactions in a 384-well format (0.5 µM in 20 µL/well), a significant reduction in cost. Moreover, the FP protocol and FRAM-based assays are HTS-compatible mix-and-measure assays that routinely yield *Z*’ factor values > 0.8.

Given the importance of amylin in glucose regulation, its pronounced tendency to aggregate and cause islet dysfunction in T2DM [3], and given emerging evidence that it forms deposits in the brain in Alzheimer’s disease [4,5], there is a great need to better understand the factors regulating amylin proteostasis. The amylin degradation assays we have developed hold promise to facilitate new discoveries in this area, which we hope might lead to novel therapeutics for T2DM and other conditions.

## Figures and Tables

**Table 1:** Sequences of the amylin substrates tested in this study and problems where encountered.

Name	Sequence ^1^	Problem
Substrate 1	5-FAM-KCNTATCATQRLANFLVHSSNNFGAILSSTNVGSNTY- [Lys(Ahx-biotin)]-amide	biotin does not bind to avidin
Substrate 2	[Lys(Ahx-biotin)]- CNTATCATQRLANFLVRSSNNLGPVLPPTNVGSNT- [Lys(FITC)]-amide	biotin does not bind to avidin
Substrate 3 (FrAB)	5-FAM-KCNTATCATQRLANFLVRSSNNLGPVLPPTNVGSNTY-[Lys(Ahx-biotin)]-amide	--
Substrate 4 (FRAM)	KCNTATCATQR[Lys(DABCYL)]ANFLVR[Glu(EDANS)]-SNNLGPVLPPTNVGSNTY	utility may be limited to IDE

^1^ All substrates were synthesized with a disulfide bond between Cys2 and Cys7. Ahx = aminohexanoic acid; DABCYL = 4-(dimethylaminoazo)benzene-4-carboxylic acid; EDANS = 5-((2-aminoethyl)amino)naphthalene-1-sulfonic acid; FAM = fluorescein amidite; FITC = fluorescein isothiocyanate.
